# Cecal Microbiota in Broilers Fed with Prebiotics

**DOI:** 10.3389/fgene.2017.00153

**Published:** 2017-10-17

**Authors:** Dany Mesa, Daniel R. Lammel, Eduardo Balsanelli, Claudia Sena, Miguel D. Noseda, Luiz F. Caron, Leonardo M. Cruz, Fabio O. Pedrosa, Emanuel M. Souza

**Affiliations:** ^1^Department of Biochemistry and Molecular Biology, Federal University of Paraná, Curitiba, Brazil; ^2^Department of Soils and Agricultural Engineer, Federal University of Paraná, Curitiba, Brazil; ^3^Department of Pathology, Federal University of Paraná, Curitiba, Brazil

**Keywords:** mannan-oligosaccharides, chickens, gut microbiota, 16S rRNA, lactobacillus

## Introduction

Prebiotics are typically fibrous compounds that pass undigested through the upper part of the gastrointestinal tract and stimulate the growth or activity of advantageous bacteria that colonize the bowel and contribute to the well-being of their host (Gibson and Roberfroid, 1995). Some of the most widely used prebiotics in the poultry industry are fructo-oligosaccharides, mannan-oligosaccharides, galacto-oligosaccharides and beta-glucans (Huyghebaert et al., 2011).

Selective fermentation of some prebiotics has been shown to induce changes in the composition and/or activity of the gastrointestinal microbiota, improving the health of the host (Gibson et al., 2004). Zhenping et al. (2013) showed increased growth performance, enhanced endocrine metabolism, and improved immune function in broiler chickens after in-feed supplementation with xylo-oligosaccharides prebiotics. Moreover, changes in enteric bacteria in the cecum (Spring et al., 2000) and improved intestinal morphology have been observed in broilers fed with dietary mannan-oligosaccharide (Baurhoo et al., 2009).

Because the microbiota can be modified (Khoruts et al., 2010; Borody and Khoruts, 2012) it constitutes an attractive target for therapeutic manipulation. However, successful outcome of such manipulations require a better understanding of the interactions between the host and its microbiota (Hamilton et al., 2013; Van Nood et al., 2013).

The composition and diversity of chicken intestinal microbiota were previously investigated using cultivation-based methodologies (Fernandez et al., 2002; Cross et al., 2007). However, the use of DNA-based molecular biology techniques, such as metagenomics and new generation DNA sequencing, allowed new opportunities to characterize uncultivable members of intestinal microbiota (Gong et al., 2002) shedding light on the composition and temporal spatial location of the microbial population in broiler's intestine. Nevertheless, the knowledge of the structure, interactions and functions of the intestinal microbiota is still limited and fragmented (Oakley and Kogut, 2016).

In this work the effect of dietary supplement with prebiotics derived from yeast wall (mannan-oligosaccharide and nucleotide) on the cecal microbiota of broilers was evaluated by the massive parallel sequencing of the *16S rRNA* gene.

## Materials and methods

### Experimental design and sampling

Thirty female newborn chicks (Hubbart®) were placed on wood shavings litter for 35 days. Water and feed were given *ad libitum*. At arrival, chicks were randomly divided in groups for three different types of treatment (10 birds per treatment): treatment without additives in the feed (Neg), treatment with the prebiotics mannan-oligosaccharide (Mos), or nucleotides (Nuc). 200 g/ton of each prebiotic were incorporated in the ration from the first day until the 35th day. Briefly, the prebiotics used in this work were extracted from biomass derived from the sugar and beer industry. These by-products contain abundant in *Saccharomyces cerevisiae* yeast. The raw-matter was treated by industrial autolysis, obtaining two fractions. The first, composed mainly for yeast wall, was rich in mannan-oligosaccharides. The second fraction contained soluble yeast extract and was abundant in nucleotides (Chaud and Sgarbieri, 2006). All animal procedures were approved by the Animal Experimentation Ethics Committee of the Federal University of Paraná (authorization CEUA-Bio UFPR 898/2015). At 14 and 35 days of age, the chicks' body weights were recorded and samples were collected for DNA extraction, purification and sequencing. Immediately after euthanasia, the abdominal cavity was exposed and the cecum was dissected from the other intestinal sections. The cecum from each bird was cut open, and the contents were collected in a sterile 2-ml tube, stored on ice and later frozen and stored at −80°C until use. In total, 24 samples for cecal contents were collected (four chickens per treatment at 14 and 35 days of age).

### DNA extraction, *16S rRNA* gene amplification and sequencing

Genomic DNA from each sample was isolated from 200 mg of cecal luminal content using PowerFecal® DNA Isolation Kit (MO BIO laboratories, Inc.). The variable V4 region of *16S rRNA* gene was amplified using the universal primers 515F and 806R (Caporaso et al., 2011) and KlenTaq Master Mix (Sigma). The PCR conditions used were 94°C for 3 min; 18 cycles of 94°C for 45 s, 50°C for 30 s and 68°C for 60 s; followed by 72°C for 10 min. The amplicons were quantified with Qubit using HS dsDNA kit (Invitrogen), diluted to 500pM and pooled. Then, 16pM of pooled DNA were sequenced using MiSeq reagent 500V2. Sequencing was performed using an Illumina MiSeq® sequencer (Illumina) obtaining paired-end reads of 250 bp as described (Caporaso et al., 2011). The dataset were submitted in NCBI site under the BioSample accession code SAMN07211773 (http://www.ncbi.nlm.nih.gov/biosample/7211773).

### Diversity analysis

Sequencing data were analyzed with the QIIME pipeline (Caporaso et al., 2010). Since the Illumina output ranged from approximately 40,000 to 172,499 reads, the read number was re-sampled to 41,800 reads per sample, allowing for the diversity comparisons. Sequences were quality filtered and identified at the phyla and genus levels using the open-reference OTU method implemented in QIIME and the SILVA database (123 release) (Yilmaz et al., 2013; Rideout et al., 2014). Basic diversity analysis (OTU number, Unifrac-distances and cluster by Neighbor-Joining method) was conducted using QIIME, and the OTU table exported to R for further analyses.

### Statistical analysis

Statistical analyses were performed using the Stats package included in RStudio software (RStudio-Team, 2015). First, data was explored using multivariate tools. Weighted unifrac-distances previously obtained from QIIME were used to sample ordination using PCoA and cluster analyzes. Since the samples showed a non-parametric distribution by the Shapiro Wilk test, the genera previously identified were compared according to the prebiotic treatments using the Kruskal Walli's test. Only statically significant results were reported (*P* < 0.05).

## Results

The use of the prebiotics Mos and Nuc resulted in no changes in the body weight of the birds in 14 or 35 days (not shown). However, they did cause several changes in the gut microbiota. Using multivariate analysis, small changes in the microbiota were observed at 14 days, but differences were clearer after 35 days (Table 1). At 14 days, *Firmicutes* was the most predominant phylum in all treatments and, on average, accounted for 84.5% of identified sequences, followed by *Proteobacteria* (7.9%) and *Tenericutes* (4.3%).

**Table 1:** Most prevalent bacterial genera in broiler's cecum microbiota.

**Genera**	**Treatment**	**14 day**	**K.W**.	**35 day**	**K.W**.	**Time**
*Faecalibacterium*	Neg	21.5 ± 3.8	A	6.1 ± 1.0	A	^*^
	Mos	25.7 ± 2.9	A	1.7 ± 0.5	B	^*^
	Nuc	13.6 ± 3.6	B	1.3 ± 0.1	B	^*^
*Bacteroides*	Neg	0		10.9 ± 1.4	B	^*^
	Mos	0		17.7 ± 4.5	B	^*^
	Nuc	0		35.9 ± 1.9	A	^*^
*Lactobacillus*	Neg	8.4 ± 2.2	A	3.4 ± 0.9	B	^*^
	Mos	8.6 ± 2.0	A	3.5 ± 1.2	B	^*^
	Nuc	2.9 ± 0.7	B	6.8 ± 1.1	A	^*^
*Streptococcus*	Neg	9.7 ± 1.2	A	3.3 ± 1.4	B	^*^
	Mos	4.0 ± 3.8	B	5.9 ± 0.4	A	^*^
	Nuc	1.9 ± 1.7	B	5.6 ± 0.8	A	^*^
*Lachnoclostridium*	Neg	5.4 ± 1.2		3.1 ± 0.5	A	^*^
	Mos	4.3 ± 0.3		1.6 ± 0.3	B	^*^
	Nuc	5.2 ± 1.2		3.0 ± 0.1	A	^*^
*Anaerotruncus*	Neg	1.0 ± 0.2	B	6.3 ± 0.3	A	^*^
	Mos	1.7 ± 0.3	A	3.5 ± 1.0	B	^*^
	Nuc	2.2 ± 0.4	A	5.9 ± 0.3	A	^*^
*Ruminiclostridium* 5	Neg	3.2 ± 0.3	C	2.4 ± 0.1	A	
	Mos	5.1 ± 0.8	B	0.9 ± 0.3	B	^*^
	Nuc	6.5 ± 1.6	A	0.8 ± 0.1	B	^*^
*Subdoligranulum*	Neg	7.2 ± 2.3	A	1.6 ± 0.2	A	^*^
	Mos	1.5 ± 0.4	B	0.4 ± 0.1	B	
	Nuc	7.5 ± 2.9	A	0.1 ± 0.0	B	^*^
*Parabacteroides*	Neg	0		0	A	
	Mos	0		11.4 ± 4.4	B	^*^
	Nuc	0		0	B	
*Escherichia-Shigella*	Neg	4.2 ± 1.5	A	0.4 ± 0.1	A	^*^
	Mos	3.4 ± 2.0	A	0.2 ± 0.1	B	^*^
	Nuc	2.7 ± 2.5	B	0.1 ± 0.0	B	^*^
*Alistipes*	Neg	0		4.9 ± 0.4	A	^*^
	Mos	0		4.1 ± 1.2	A	^*^
	Nuc	0		0.9 ± 0.2	B	^*^

Data is shown by treatment (%, mean and standard error, n = 4). The K.W. columns show the result of statistical comparison between treatments (Neg, Mos, and Nuc) for each genus by the Kruskal Wallis test. Same uppercase letters indicate no statistical difference in genus abundance between treatments. The column Time shows statistical comparison between the two time points by the Tukey test. Asterisk (^*^) indicates significant difference between sampling times (P < 0.05). Control group (Neg), mannan-oligosaccharide (Mos) and nucleotide (Nuc).

After 35 days, *Firmicutes* remained the most predominant phylum in cecum in all treatments and, on average, accounted for 61.4% of all the bacterial sequences, followed by *Bacteroidetes* (29%), *Proteobacteria* (4.5%), and *Tenericutes* (4.2%). Interestingly, the phylum *Bacteroidetes* was only detected at 35 days (Supplementary Material 1). The prevalence of *Firmicutes* at 14 days might be explained by the higher need of butyrate by the young chicken growing intestine (Van der Wielen et al., 2000). Since in adult chickens maximal butyrate production is no longer required, digestion of complex polysaccharides by representatives of *Bacteroidetes* results in the production of both propionate and butyrate, which might be a more advantageous balance of nutrients (Polansky et al., 2016). In agreement with this assumption, Polansky et al. (2016) observed a gradual increase of *Bacteroidetes* counts in the cecum from the third week of life of chickens.

By further dissecting the 16S ribosomal sequences, a total of 665 OTUs corresponding to bacterial genera were detected, and were identified using the SILVA database (release 123) (Supplementary Material 2). Among all identified bacterial genera in the cecal microbiota, the most abundant in all treatments were *Faecalibacterium, Bacteroides, Lactobacillus, Streptococcus, Lachnoclostridium, Anaerotruncus, Ruminiclostridium* 5*, Subdoligranulum, Parabacteroides, Escherichia-Shigella*, and *Alistipes* (Supplementary Material 2). At 14 days, the most prevalent genera in all groups were *Faecalibacterium* accounting for, on average, 20.3% of all cecal bacterial sequences. The second most prevalent genera was *Lactobacillus* with 6.65% of bacterial sequences, followed by *Subdoligranulum* (5.42%) (Table 1). At 35 days, *Bacteroides* was the most predominant genera in all treatments, accounting for, on average, 21.5% of the cecal sequences. The second most prevalent was *Anaerotruncus* (5.59%), followed by *Streptococcus* (4.93%). Besides, at 35 days, the frequencies of 130 genera significantly differed between treatments. Over time an increase in the count of sequences was observed in *Bacteroides, Anaerotruncus*, and *Alistipes* genera. Interestingly, the *Parabacteroides* genus increased at 35 days only in the Mos treated group, while the genera *Faecalibacterium, Lachnoclostridium, Ruminiclostridium* 5, and *Subdoligranulum* decreased at 35 days (Supplementary Material 3).

**Figure 1:**
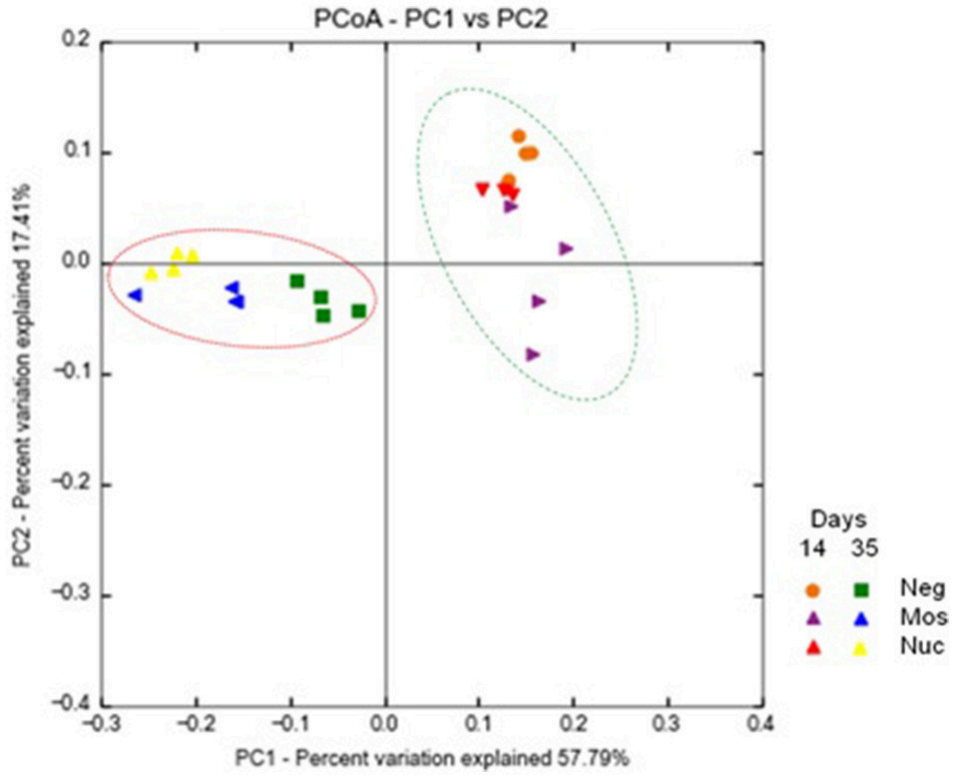
Principal Component Analysis (PCoA) of broiler's cecal bacterial community fed with yeast-derived prebiotics. Control group (Neg) mannan-oligosaccharide (Mos) and nucleotide (Nuc) are shown. Samples were compared according to the out composition at the genus level as identified by the SILVA database (SILVA 123 release) using the Unifrac distance weighted method.

Moreover, Principal Component Analysis of OTUs representation clearly showed the age explained most of the differences (57.8%), followed by treatment (17.4%) (Figure 1). This result was also observed in cluster analysis of microbial community by the Neighbor-Joining method (Supplementary Material 4).

The use of Nuc as prebiotics significantly increased the count of sequences of *Lactobacillus* and significantly decreased the count of sequences of *Escherichia-Shigella* at 35 day. On the other hand, the use of prebiotic Mos significantly increased the count of sequences of *Butyricimonas* and *Roseburia* genera at 35 day (Supplementary Material 6). The supplementation with both prebiotics significantly decreased the *Chao*1 index at 35 days, when compared to the negative group. This index is a species richness estimator, which estimates the total number of species present in a community (Supplementary Material 5).

## Conclusion

In conclusion the composition of cecal microbiota is not constant and develops over time in chickens. Moreover, prebiotic supplementation significantly affected the microbial community structure in the cecum in broiler chickens with most significant shifts at 35 days. Although the prebiotics Mos and Nuc caused a decrease the richness and the diversity in the cecum, the prevalence of beneficial bacteria increased in both treatments. Nuc increased the count of sequences of *Lactobacillus* and decreased the count of sequences of *Escherichia-Shigella*, while Mos significantly increased the count of sequences of *Butyricimonas* and *Roseburia*. The *Lactobacillus, Butyricimonas*, and *Roseburia* genera are known for producing short-chain fatty acids (SCFAs) and have shown beneficial effects on the host's development and health (Pryde et al., 2002; Farnworth, 2005; Jhangi et al., 2014; Loman and Tappenden, 2016).

## Author contributions

MN provided the prebiotics. DM and DL analyzed the data and performed the numerical and statistical analyses. DM and ES designed the work and wrote the manuscript. DL and LMC processed the sequence raw data. CS and LFC designed the protocol. DM performed DA extraction from samples. EB performed amplicons library preparation and sequencing. FP and ES supervised the work.

### Conflict of interest statement

The authors declare that the research was conducted in the absence of any commercial or financial relationships that could be construed as a potential conflict of interest.
