# Supplementary material for: Cecal Microbiota in Broilers Fed with Prebiotics
Source: Front Genet. 2017 Oct 17;8:153. doi: 10.3389/fgene.2017.00153 (PMC5650999; doi:10.3389/fgene.2017.00153)
Supplement: Supplementary file 3 [file DataSheet3.pdf]

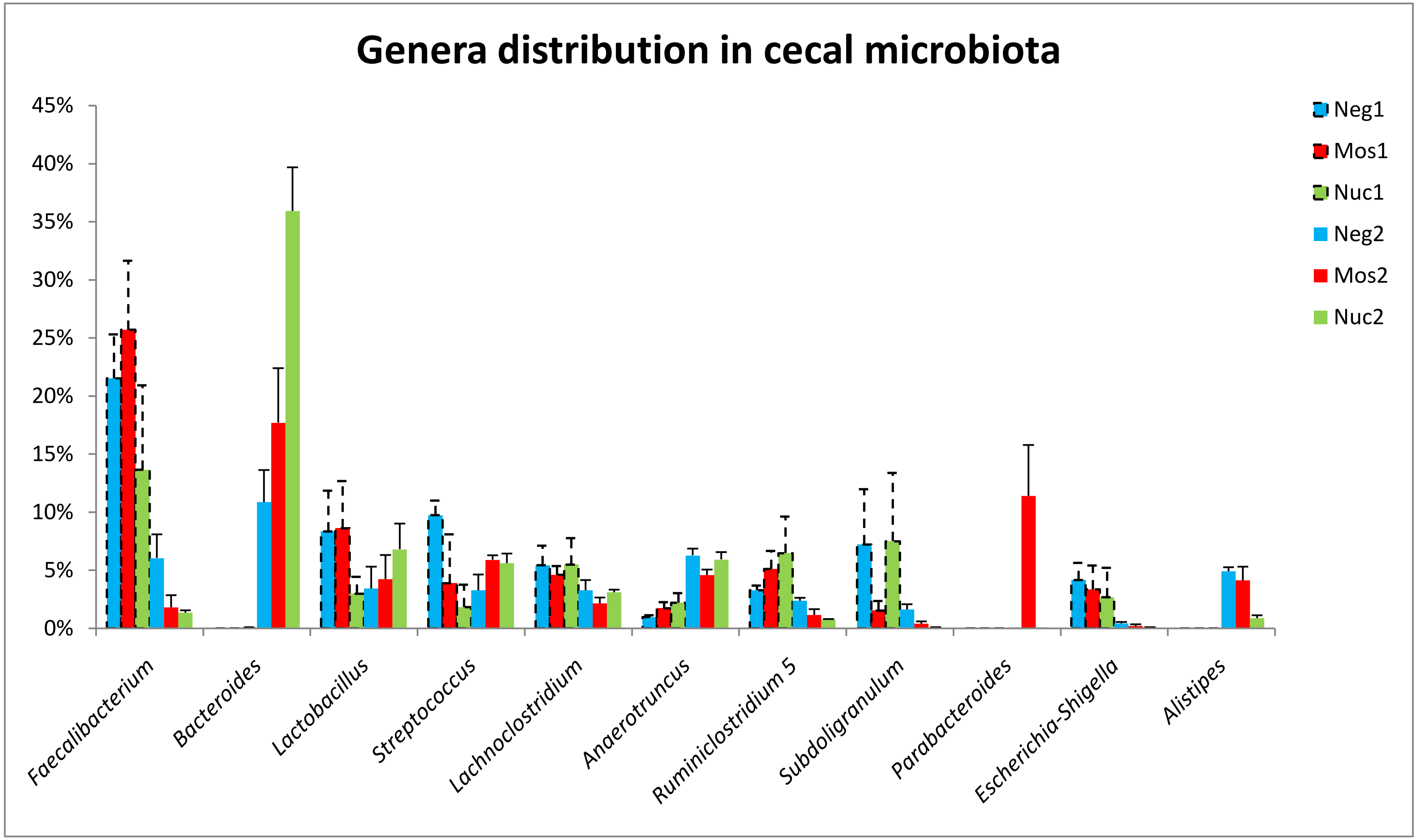

Supplementary Material 3. Distribution of the most abundant genera in the cecum. Control group (Neg), mannan-oligosaccharide (Mos) and nucleotide (Nuc). Number “1” means at 14 day and number “2” at 35 day. Data is show by treatment (%, mean and standard error, n=4).
