# Supplementary material for: Cecal Microbiota in Broilers Fed with Prebiotics
Source: Front Genet. 2017 Oct 17;8:153. doi: 10.3389/fgene.2017.00153 (PMC5650999; doi:10.3389/fgene.2017.00153)
Supplement: Supplementary file 4 [file DataSheet4.pdf]

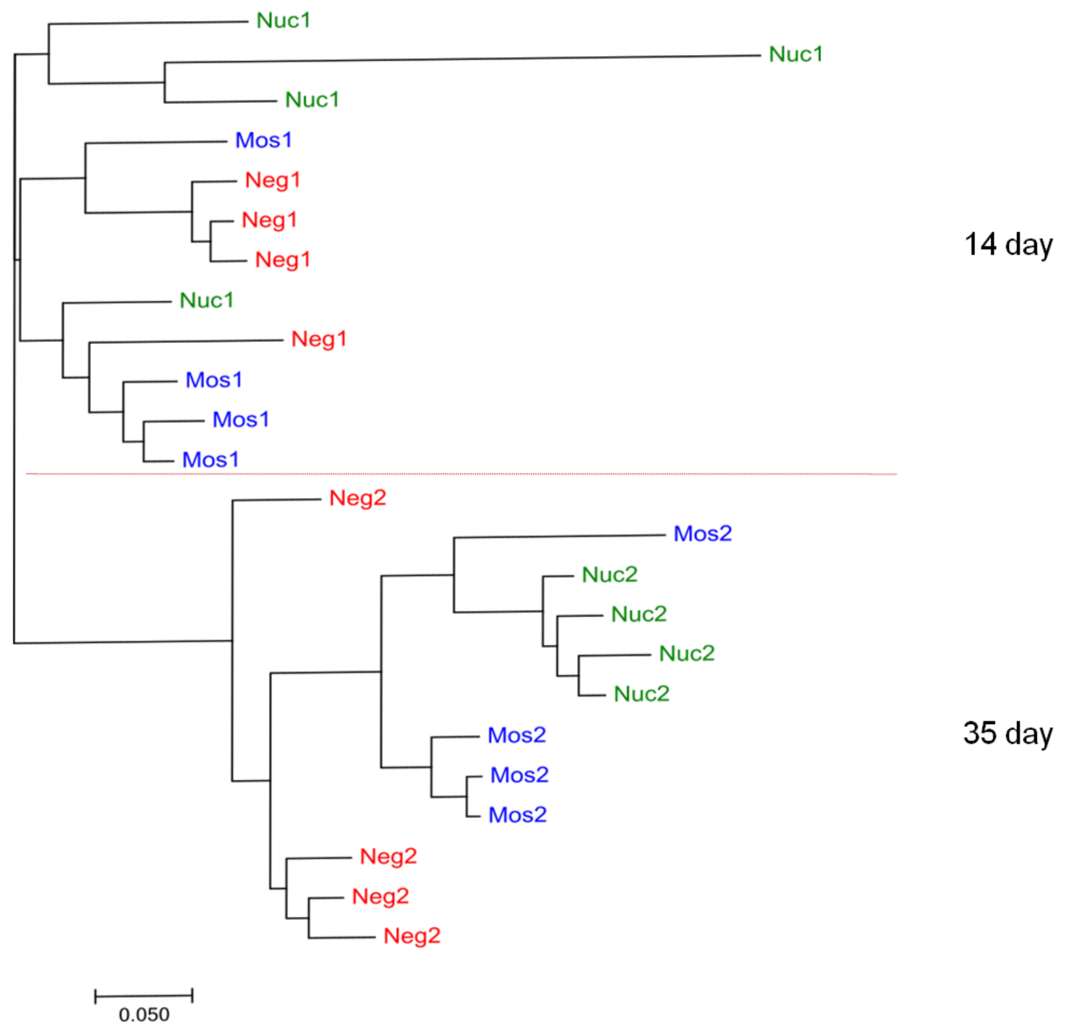

Supplementary Material 4. Cluster of distance between groups by a Neighbor-joining method. Control group (Neg) mannan-oligosaccharide (Mos) and nucleotide (Nuc). Samples were compared according to the OTUs diversity at the genus level with (Silva 123 release) database using the Unifrac distance weighted.
