# Supplementary material for: Cecal Microbiota in Broilers Fed with Prebiotics
Source: Front Genet. 2017 Oct 17;8:153. doi: 10.3389/fgene.2017.00153 (PMC5650999; doi:10.3389/fgene.2017.00153)
Supplement: Supplementary file 5 [file DataSheet5.pdf]

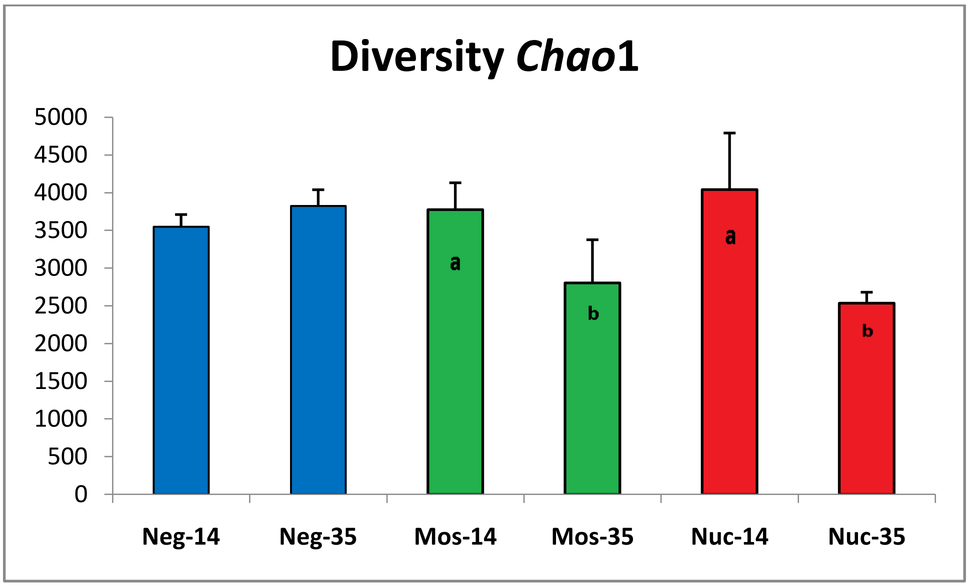

Supplementary Material 5. Chao1 index of different bacterial groups in cecum at 14 and 35 days. Different letters mean statistical difference in genera abundance between sampling times by the Tukey test *(P*<0.05). Data is shown by treatment (%, mean and standard error, n=4). Control group (Neg), mannan-oligosaccharide (Mos) and nucleotide (Nuc).
