# Supplementary material for: Cecal Microbiota in Broilers Fed with Prebiotics
Source: Front Genet. 2017 Oct 17;8:153. doi: 10.3389/fgene.2017.00153 (PMC5650999; doi:10.3389/fgene.2017.00153)
Supplement: Supplementary file 6 [file DataSheet6.pdf]

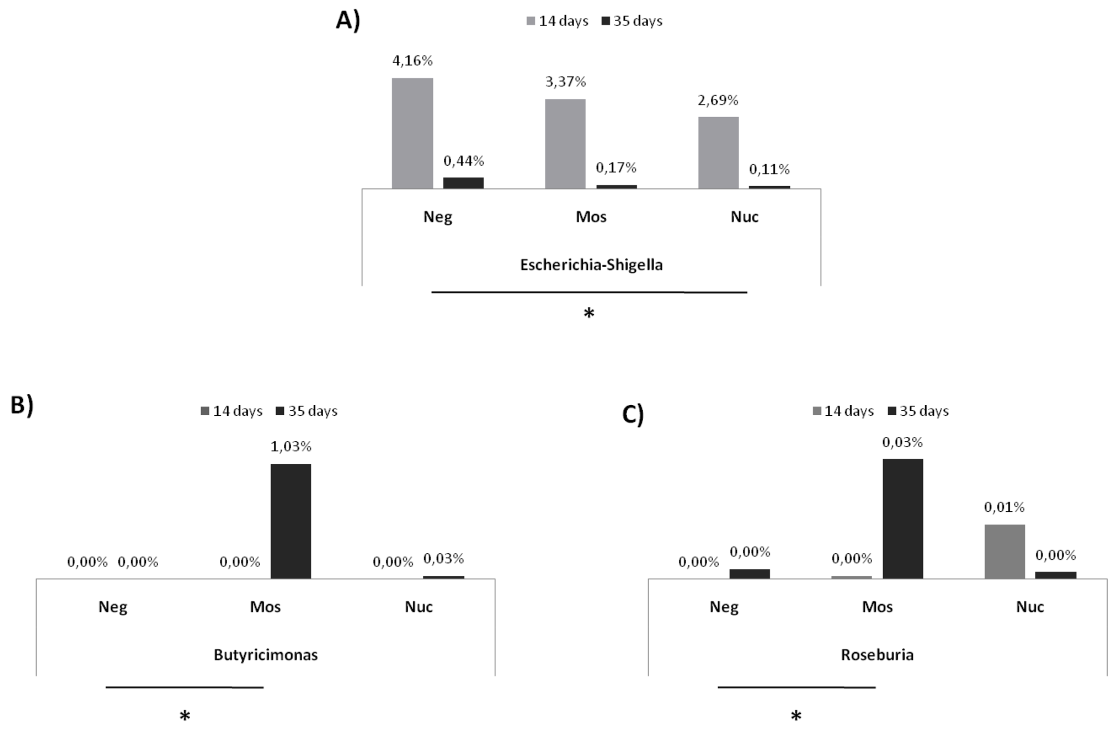

Supplementary Material 6. A). Nuc as prebiotic decreased significantly the count of sequences of *Escherichia-Shigella*. B). Mos as prebiotic increased significantly the count of sequences of *Butyricimonas* and *Roseburia* (C).
